# Editorial—Self-assembly and emergent properties in populations of microbiologists

**DOI:** 10.1038/s43705-022-00150-3

**Published:** 2022-07-26

**Authors:** Federico M. Lauro, Michael J. Manefield

**Affiliations:** 1 Asian School of the Environment, Nanyang Technological University, Singapore, 639798 Singapore; 2 Singapore Centre for Environmental Life Sciences Engineering, Nanyang Technological University, Singapore, 637551 Singapore; 3 School of Civil and Environmental Engineering, UNSW Sydney, Sydney, NSW 2052 Australia

**Keywords:** Microbial ecology, Food webs

Over a decade ago, a clade of microbiologists gathered informally in a back room at the Australian Museum in Sydney to feed on pizza and beer and listen to three short talks from local microbiologists. Sydney, Australia has five Universities with strong microbiology research and teaching programs, but at the time there was limited interaction and syntrophy between them. We wanted to put them together in a flask and shake.

Akin to a freshly inoculated culture, over successive monthly gatherings, the crowd grew exponentially, and the Joint Academic Microbiology Seminars (JAMS) were born. JAMS nodes have now been established in Sydney, Adelaide and Brisbane in Australia, Kuala Lumpur in Malaysia and in Singapore. A holobiont that includes a venue and commensal students and early career microbiologists, assembles in these cities as much to socialise as to share their research, learn from others, brainstorm, develop professional skills and grow their hyphal networks.

A decade ago, JAMS also started holding annual symposia knitting the Australasian microbiology clades together with world class speaker line-ups and lashings of fun. In 2021, this culminated in JAMS10, our 10th annual symposium. Despite pandemic lockdowns forcing JAMS10 online, it was an unmitigated success with our best line-up and highest attendance ever. This special issue celebrates what amounts to a game-changing (mould-breaking) movement in how microbiologists interact globally.

JAMS gets its energy from scientific youth and passion. Their enthusiasm is boundless and when you hand students and early career researchers the reigns you slough off burdensome tradition and welcome innovation. This is behind the fusion of social and professional activity that enables JAMS to expand under its own steam. When someone puts their hand up with a crazy idea, they are encouraged to run with it. JAMS has always been a safe place to experiment with how we interact. We have always found, perhaps unsurprisingly, that microbiologists, like the microbes they study, are social creatures and that they relate better to a good time at events that can be humorously rough around the edges than they do to slick and sterile professional conference organisation. Microbiologists shine brightest when they are relaxed and operating on a shoestring enables events to be free or bargain basement cheap facilitating attendance by the budding future caretakers of our knowledge.

JAMS is so much more than the sum of its constituents. In a decade, JAMS has put thousands of bums on seats and hundreds of speakers in the spotlight, embracing the diverse microbial world and creating hundreds of opportunities for students and young researchers and educators to develop professional skills from presenting, debating, chairing, hosting and organising events and serving on committees as treasurers, secretaries and directors. The connections created and research findings disseminated by JAMS are countless. The gains in reputation that have grown through JAMS are invaluable in early careers where opportunities are few and far between as older more established academics consume all available electron acceptors.

Whilst we have little to do with running JAMS these days, we are immensely proud of everyone who has contributed and everything it has become. We look forward to watching the garden grow larger and wilder into the future. To all the students and ECRs out there, this is your world to inherit, and you can make of it what you will. JAMS exists for you, so feel free to reach out to learn how you can contribute and benefit. Like a planktonic cell choosing a surface to colonise, JAMS nodes can spring up anywhere. And like biofilms, that can often be beneficial or moderately annoying, the only thing you cannot do is ignore them.

